# Comparative outcomes of arteriovenous accesses with and without tunneled hemodialysis catheters: A retrospective cohort study

**DOI:** 10.1097/MD.0000000000051077

**Published:** 2026-10-16

**Authors:** Heng Su, Ting-Wei Lin, Jian-Han Chen, Hsuan-Yin Wu

**Affiliations:** a Division of Cardiovascular Surgery, Department of Surgery, E-DA hospital and College of Medicine, I-Shou University, Kaohsiung, Taiwan; b Division of General Surgery, E-Da Hospital, Kaohsiung, Taiwan; c Department of Surgery, Bariatric and Metabolism International Surgery Center, E-Da Hospital, Kaohsiung, Taiwan; d School of Medicine, College of Medicine, I-Shou University, Kaohsiung, Taiwan.

**Keywords:** arteriovenous fistula, arteriovenous graft, hemodialysis, patency, tunneled hemodialysis catheter, vascular access

## Abstract

This study compared patency rates of arteriovenous accesses with and without concurrent tunneled hemodialysis catheter (THC) use, addressing limited research on combined outcomes. A retrospective cohort analysis was conducted on patients undergoing arteriovenous access creation at a single hospital between January 2021 and December 2022. Primary outcomes were the 6-month primary patency rate and functional dialysis success. Secondary outcomes were the factors associated with each of these endpoints. Statistical analyses employed *t* tests, chi-square tests, logistic regression, and Kaplan–Meier survival curves. A total of 319 vascular accesses were included. The 6-month patency rate was significantly higher in the access-only group than in the access + THC group (87.04% vs 78.20%, *P* = .04). THC usage (odds ratio [OR] = 0.49, 95% confidence interval [CI]: 0.25–0.95, *P* = .03), graft usage (OR = 0.38, 95% CI: 0.18–0.82, *P* = .01), and higher hemoglobin levels (OR = 0.80, 95% CI: 0.65–0.96, *P* = .02) were associated with reduced patency rates. Functional dialysis success at 6 months did not differ significantly between groups (87.96% vs 83.89%, *P* = .21). However, older age (OR = 0.96, 95% CI: 0.93–0.99, *P* = .01), use of a cubital artery to an autologous vein (OR = 0.39, 95% CI: 0.19–0.82, *P* = .01) or graft (OR = 0.40, 95% CI: 0.17–0.97, *P* = .04), and higher hemoglobin levels (OR = 0.81, 95% CI: 0.66–0.99, *P* = .03) were associated with a lower likelihood of functional dialysis success. Concurrent THC use was independently associated with reduced 6-month primary patency without a corresponding reduction in the short-term delivery of dialysis, suggesting an increased need for interventions to maintain the access. Judicious use of THCs and careful consideration of the vascular access type are important to optimize vascular access outcomes in patients undergoing hemodialysis.

## 1. Introduction

Hemodialysis (HD) serves as a critical lifeline for patients with renal failure,^[1]^ ensuring the effective removal of waste products from the blood when the kidneys are compromised. This is significant in regions such as Taiwan, known for having one of the highest rates of patients on dialysis.^[2]^ In 2020, chronic kidney diseases (CKD), acute kidney failure, and other kidney-associated treatments consumed 7.2% of the overall budget of Taiwan’s national healthcare insurance, having the highest cost among all treatments for other diseases. Furthermore, more than 4,45,000 patients with kidney diseases drained an overall 56,245 million New Taiwan Dollars (NTD), averaging 1,26,417 NTD per patient per year.^[3]^

The National Kidney Foundation Kidney Disease Outcomes Quality Initiative guidelines recommend that referral for dialysis access creation should be made in patients with CKD having an estimated glomerular filtration rate (eGFR) of 15 to 20 mL/min/1.73 m^2^, and the European Society for Vascular Surgery recommends that permanent vascular access should be created 3–6 months before the expected start of HD treatment.^[4,5]^ The establishment of reliable vascular access is essential to successful hemodialysis.^[6]^ Typically, this is achieved through the creation of an arteriovenous fistula (AVF), commonly referred to as a shunt. When the patient’s vascular quality is incapable of creating a shunt, and an artificial graft is usually then used to connect the artery and vein, thereby allowing direct puncture on the artificial graft during HD when the arteriovenous graft (AVG) is ready to use. In certain cases, alongside the AVF or AVG, a tunneled HD catheter (THC) is also placed to facilitate dialysis while the access matures to a dialysis-acceptable size.^[7]^ Generally, THCs are regarded as a bridge access to AVF or AVG;^[8,9]^ however, studies have discouraged the use of THCs as the first choice of permanent vascular access because of the higher risk of complications and shorter expected service life, mostly due to recurrent infections.^[10,11]^

The patency, or the open and unobstructed nature of these shunts, plays a pivotal role, directly influencing the efficacy of HD.^[12]^ Although previous studies have extensively explored the roles and benefits of AVFs and THCs individually in HD,^[13,14]^ a notable gap remains in the literature regarding the comparative outcomes of these 2 approaches when used simultaneously. Specifically, the effect of simultaneous AVF and THC placement on AVF patency rates has not been thoroughly investigated. Given the critical importance of shunt patency in ensuring effective HD, understanding the potential advantages or challenges of this combined approach is paramount.

This study seeks to bridge this research gap by directly comparing the patency rates of AVFs in 2 distinct groups: those receiving only an AVF and those receiving an AVF with a THC. Through this exploration, we aim to provide clearer guidance for clinicians in optimizing vascular access strategies for patients undergoing HD.

## 2. Materials and methods

### 2.1. Data sources and research samples

We retrospectively analyzed data from a comprehensive computerized vascular access database of patients who underwent AVF creation for HD access at E-Da Hospital between January 2021 and December 2022. This study was approved by the Institutional Review Board of E-Da Hospital (approval number EMRP-113-061). Owing to the retrospective design and the use of de-identified data obtained from existing medical records, the requirement for written informed consent was waived by the Institutional Review Board. All procedures were conducted in accordance with the Declaration of Helsinki.

### 2.2. Study population and exclusion criteria

The study included adult patients (aged ≥ 18 years) with end-stage renal disease who underwent the creation of a new AVF for HD access. Patients were categorized into 2 groups: those who had a new AVF created without a THC and those who had a new AVF with a THC inserted simultaneously. The exclusion criteria were as follows: not initiating HD within 6 months of AVF creation, undergoing a redo of a previously placed THC, being under peritoneal dialysis or having a history of peritoneal dialysis, having incomplete medical records or follow-up data (missing > 20% of the required data points), having active systemic infections and malignancies, being pregnant or breastfeeding during the study period, and having a life expectancy of <6 months because of nonrenal causes. To assess the inflammatory status and hemodynamic condition of the patients, the following parameters were evaluated within 1 week before AVF creation: the total white blood cell count (WBC), hemoglobin (Hb), and eGFR.

The decision to place a THC was not randomized. At our institution, a THC was inserted concurrently with access creation when hemodialysis had to be initiated before the access could mature, typically because of uremic symptoms, refractory volume overload, or hyperkalemia at the time of referral. Accordingly, allocation to the THC group reflected clinical urgency rather than chance.

### 2.3. Outcome measurements

The primary outcomes were the 6-month primary patency rate and functional dialysis success. Patency was defined as the maintenance of adequate blood flow for hemodialysis without the need for interventions to maintain or restore patency of upper-extremity arteriovenous accesses and was assessed using clinical examination and ultrasound assessment at regular intervals (1, 3, and 6 months postsurgery). Functional dialysis success within 6 months was defined as the delivery of hemodialysis through the newly created access using 2-needle cannulation, with the prescribed blood flow rate sustained for the full prescribed session duration, without reversion to catheter-based dialysis, without premature termination of a session attributable to the access, and without unplanned intervention on the access. This outcome was evaluated by reviewing hemodialysis session records and patient charts. Secondary outcomes were the factors associated with 6-month patency and functional dialysis success, including age, sex, weight, body mass index (BMI), presence of a THC, vascular access type, comorbidities (coronary artery disease [CAD], hypertension, and diabetes mellitus), antiplatelet use, bedridden status, WBC, Hb level, and eGFR. Access-related infection within 6 months was recorded descriptively and was not subjected to multivariable modeling. Infections were diagnosed using clinical signs (erythema, swelling, tenderness, or purulent discharge), positive blood cultures, or radiographic evidence of infection.

### 2.4. Statistical analyses

Statistical analyses were performed using SPSS (IBM Corp, Armonk). Baseline patient characteristics were compared using Student’s *t* tests for continuous variables and chi-square tests for categorical variables. For continuous variables, the normality of distribution was determined using the Kolmogorov–Smirnov test, followed by either the Wilcoxon rank-sum test for non-normally distributed data or Student *t* test for normally distributed data. Differences in basic clinical characteristics were explored in contingency tables, which were then analyzed using chi-square or Fisher’s exact tests, as appropriate. Kaplan–Meier survival curves were constructed to estimate the cumulative 6-month AVF patency and functional dialysis success. Before regression analysis, collinearity diagnostics were conducted to ensure the independence of the variables. Multivariable logistic regression analysis was employed to identify factors associated with 6-month AVF patency and functional dialysis success. Odds ratios (ORs) and their corresponding 95% confidence intervals (CIs) were calculated, with a *P*-value < .05 considered statistically significant.

## 3. Results

In total, 319 vascular accesses were included in the study, with 108 (33.9%) in the AVF group and 211 (66.1%) in the AVF + THC group. Baseline characteristics were comparable between the 2 groups, except for a significantly lower eGFR in the AVF + THC group (7.88 vs 9.59 mL/min/1.73 m^2^, *P *= .03; Table 1).

**Table 1:** Baseline characteristics of patients with AVF or AVG with and without THC.

Variable	Without THC (n = 108)	With THC (n = 211)	*P*-value
Age, yr, average	64.52	64.04	.91
Weight, mean (kg)	66.77	64.82	.44
Sex (female/total)	36 (33.30%)	88 (41.70%)	.18
BMI mean	25.70	25.07	.49
CAD	26 (24.07%)	60 (28.44%)	.42
Hypertension	90 (83.33%)	191 (90.52%)	.07
DM	68 (62.96%)	137 (64.93%)	.81
Antiplatelet use	34 (31.48%)	64 (30.33%)	.89
Bedridden status	7 (6.48%)	11 (5.21%)	.62
WBC count (10^9^/L)	7.11	7.61	.06
Hb (g/dL)	9.32	9.18	.32
eGFR (mL/min/1.73 m^2^)	9.59	7.88	.03*
Infection within 6 mo	6 (5.56%)	11 (5.21%)	.33
Site of THC placement			
Contralateral	N/A	200 (94.78%)	N/A
Ipsilateral	N/A	11 (5.22%)	N/A
Vascular access selection			
Radial artery to autologous vein	71 (65.75%)	144 (68.25%)	.87
Cubital artery to autologous vein	22 (20.37%)	43 (20.38%)	.90
Graft to autologous vein	15 (13.88%)	24 (11.37%)	.65
Autologous	93 (86.11%)	187 (88.63%)	.59
AVF patency within 6 mo	94 (87.04%)	165 (78.20%)	.04
Functional dialysis success within 6 mo	95 (87.96%)	177 (83.89%)	.21

AVF = arteriovenous fistula, AVG = arteriovenous graft, BMI = body mass index, CAD = coronary artery disease, DM = diabetes mellitus, eGFR = estimated glomerular filtration rate, Hb = hemoglobin, THC = tunneled hemodialysis catheter, WBC = white blood cell.

* Significant difference.

The 6-month patency rate was significantly higher in the AVF group than in the AVF + THC group (87.04% vs 78.20%, *P* = .04; Table 1). Multivariate analysis revealed that the presence of a THC was associated with a lower likelihood of AVF patency (odds ratio [OR] 0.49, 95% CI: 0.25–0.95, *P *= .03; Table 2), whereas graft use was also associated with reduced patency compared with a radial or cubital artery to an autologous vein (OR 0.38, 95% CI: 0.18–0.82, *P *= .01). Higher Hb levels were found to be a significant predictor of reduced patency (OR 0.80, 95% CI: 0.65–0.96, *P* = .02; Table 2). The 6-month functional dialysis success was not significantly different between the 2 groups (87.96% vs 83.89%, *P* = .21; Table 1). However, multivariate analysis showed that older age (OR 0.96, 95% CI: 0.93–0.99, *P *= .01), use of a cubital artery to an autologous vein (OR 0.39, 95% CI: 0.19–0.82, *P *= .01) or a graft (OR 0.40, 95% CI: 0.17–0.97, *P* = .04), and higher Hb levels (OR 0.81, 95% CI: 0.66–0.99, *P *= .03) were associated with a lower likelihood of functional dialysis success (Table 3). Access-related infection within 6 months occurred in 6 of 108 accesses (5.56%) in the group without a THC and in 11 of 211 accesses (5.21%) in the group with a THC, with no significant difference between groups (*P* = .33; Table 1).

**Table 2:** Univariate and multivariate analyses for factors associated with AVF or AVG patency within 6 months.

Variables	Univariate		Multivariate	
	OR (95% CI)	*P*-value	OR (95% CI)	*P*-value
Age (yr)				
≦70	1.00 (reference)			
>70	1.31 (0.69–2.50)	*P *= .41		
Sex				
Women	1.00 (reference)			
Men	0.87 (0.49–1.53)	*P *= .62		
Weight				
<60 kg	1.00 (reference)			
>60 kg	0.99 (0.89–1.01)	*P* = .75		
BMI				
<30	1.00 (reference)			
>30	0.82 (0.36–1.85)	*P* = .62		
AVF + THC	0.53 (0.31–0.78)	*P* = .05	0.49 (0.25–0.95)	*P* = .03
Vascular access selection				
Radial artery to autologous vein	1.00 (reference)			
Cubital artery to autologous vein	0.95 (0.50–2.20)	*P* = .90		
Graft to autologous vein	0.36 (0.16–0.84)	*P* < .01	0.38 (0.18–0.82)	*P* = .01
CAD	1.01 (0.98–1.21)	*P* = .95		
Hypertension	0.94 (0.88–1.12)	*P* = .97		
DM	1.36 (0.87–2.10)	*P* = .28		
Antiplatelet	1.04 (0.78–1.88)	*P* = .89		
Bedridden	1.16 (0.80–1.21)	*P* = .81		
WBC				
<10.7 (10^9^/L)	1.00 (reference)			
>10.7 (10^9^/L)	0.95 (0.38–2.43)	*P* = .92		
Hb				
<11 (g/dL)	1.00 (reference)			
>11 (g/dL)	0.78 (0.61–0.89)	*P* = .01	0.80 (0.65–0.96)	*P* = .02
eGFR				
<15 (mL/min/1.73 m^2^)	1.00 (reference)			
≧15 (mL/min/1.73 m^2^)	1.77 (0.70–4.45)	*P* = .31		

AVF = arteriovenous fistula, AVG = arteriovenous graft, BMI = body mass index, CAD = coronary artery disease, CI = confidence interval, DM = diabetes mellitus, eGFR = estimated glomerular filtration rate, Hb = hemoglobin, OR = odds ratio, THC = tunneled hemodialysis catheter, WBC = white blood cell.

**Table 3:** Univariate and multivariate analyses for factors associated with functional dialysis success within 6 months.

Variables	Univariate		Multivariate	
	OR (95% CI)	*P*-value	OR (95% CI)	*P*-value
Age (yr)				
≦70	1.00 (reference)			
>70	0.96 (0.92–0.98)	*P *= .01	0.96 (0.93–0.99)	*P* = .01
Sex				
Women	1.00 (reference)			
Men	0.75 (0.40–1.41)	*P *= .37		
Weight				
<60 kg	1.00 (reference)			
>60 kg	1.02 (0.91–1.35)	*P* = .66		
BMI				
<30	1.00 (reference)			
>30	0.62 (0.23–1.65)	*P* = .36		
AVF + THC	0.71 (0.31–0.98)	*P* = .33		
Vascular access selection				
Radial artery to autologous vein	1.00 (reference)			
Cubital artery to autologous vein	0.33 (0.19–0.89)	*P* = .03	0.39 (0.19–0.82)	*P* = .01
Graft use	0.31 (0.21–0.96)	*P* = .03	0.40 (0.17–0.97)	*P* = .04
CAD	1.67 (0.87–3.21)	*P* = .12		
Hypertension	0.74 (0.30–1.78)	*P* = .49		
DM	1.22 (0.63–2.36)	*P* = .54		
Antiplatelet	0.84 (0.42–1.67)	*P* = .62		
Bedridden	1.17 (0.32–4.20)	*P* = .81		
WBC				
<10.7 (10^9^/L)	1.00 (reference)			
>10.7 (10^9^/L)	0.78 (0.26–2.33)	*P* = .65		
Hb				
<11 (g/dL)	1.00 (reference)			
>11 (g/dL)	0.79 (0.31–0.91)	*P* = .02	0.81 (0.66–0.99)	*P* = .03
eGFR				
<15 (mL/min/1.73 m^2^)	1.00 (reference)			
≧15 (mL/min/1.73 m^2^)	1.11 (0.36–3.40)	*P* = .23		

AVF = arteriovenous fistula, AVG = arteriovenous graft, BMI = body mass index, CAD = coronary artery disease, CI = confidence interval, DM = diabetes mellitus, eGFR = estimated glomerular filtration rate, Hb = hemoglobin, OR = odds ratio, THC = tunneled hemodialysis catheter, WBC = white blood cell.

The Kaplan–Meier curves were used to compare the 6-month patency and functional dialysis success between patients with AVF alone and those with AVF + THC. The 6-month patency rate was significantly higher in the AVF group than in the AVF + THC group (87.04% vs 78.20%, *P *= .04; Fig. 1). The difference in patency rates between the 2 groups became more pronounced over time, with the AVF group maintaining a higher patency rate throughout the 6-month follow-up period. In contrast, the 6-month functional dialysis success was not significantly different between the 2 groups (87.96% vs 83.89%, *P *= .21; Fig. 2). Both groups showed a similar decline in functional dialysis success over time, with the AVF group maintaining a slightly higher rate throughout the follow-up period, although this difference did not reach statistical significance.

**Figure 1.**
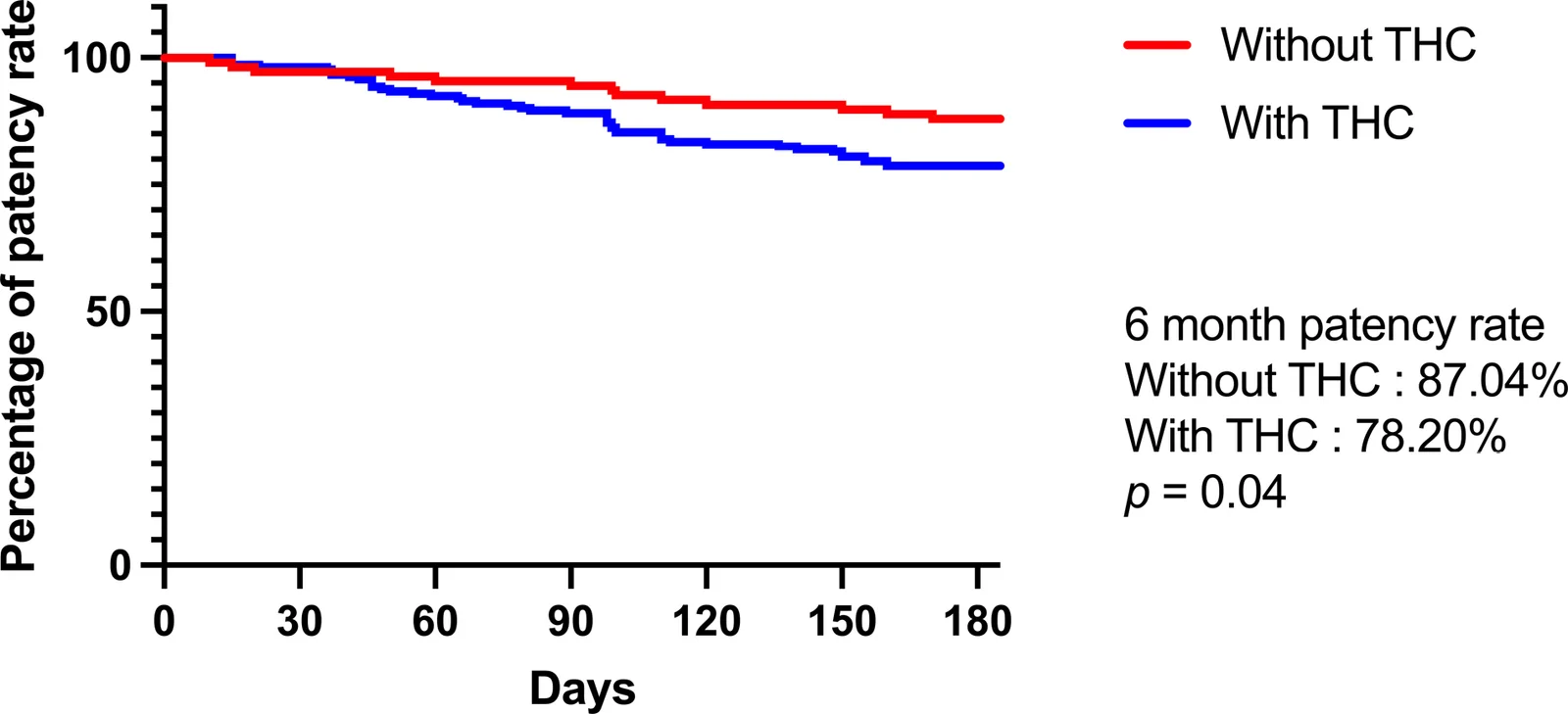
Six-month primary patency of arteriovenous accesses created with and without a concurrent tunneled hemodialysis catheter. Kaplan–Meier curves comparing primary patency within 6 months between accesses created without a concurrent tunneled hemodialysis catheter and those created with one. The 6-month primary patency rate was 87.04% without a catheter and 78.20% with a catheter (*P* = .04). THC = tunneled hemodialysis catheter.

**Figure 2.**
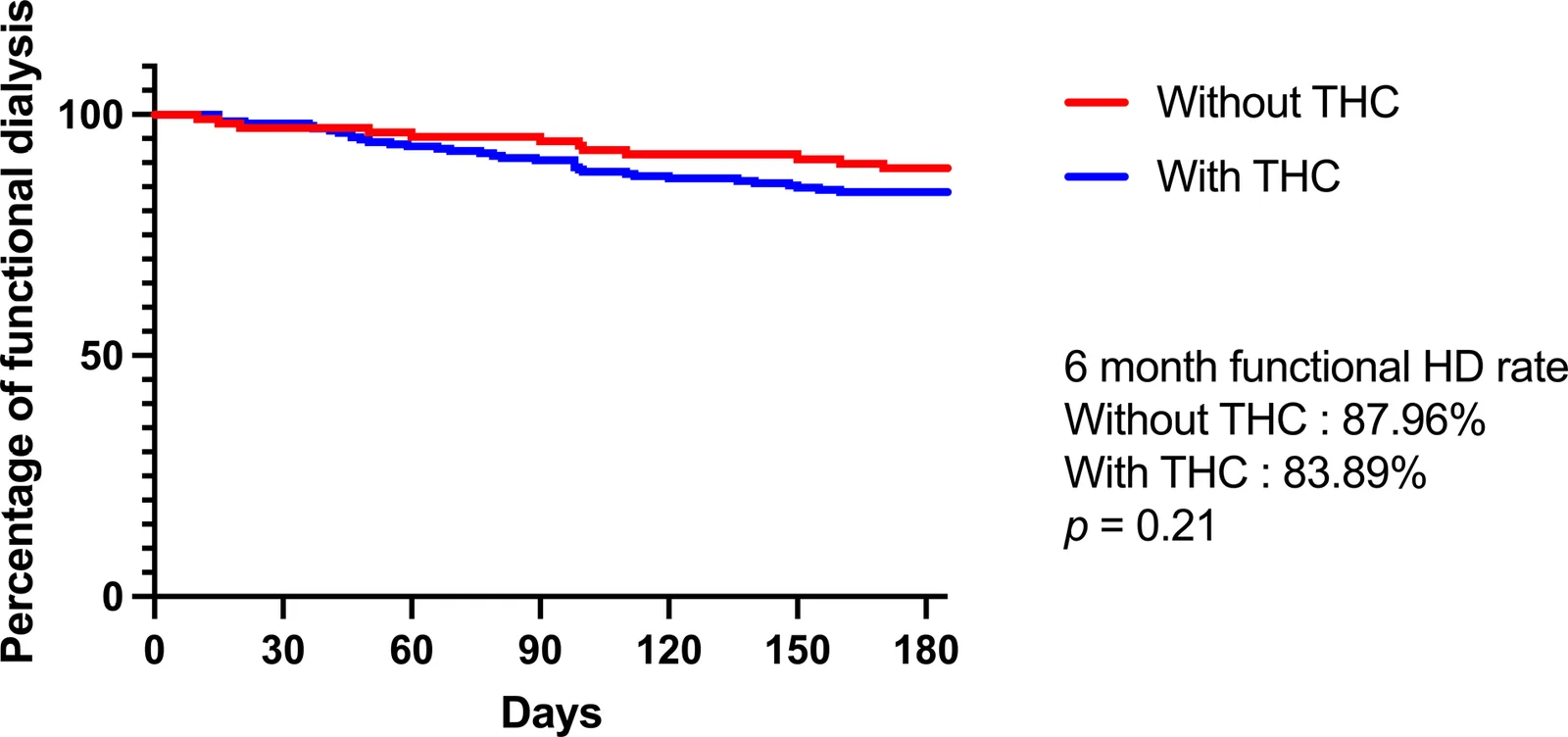
Six-month functional dialysis success of arteriovenous accesses created with and without a concurrent tunneled hemodialysis catheter. Kaplan–Meier curves comparing functional dialysis success within 6 months between accesses created without a concurrent tunneled hemodialysis catheter and those created with one. Functional dialysis success at 6 months was 87.96% without a catheter and 83.89% with a catheter (*P* = .21). HD = hemodialysis, THC = tunneled hemodialysis catheter.

## 4. Discussion

This retrospective study aimed to compare the patency rates and functional dialysis success between patients receiving only an AVF or AVG and those receiving AVF + THC or AVG + THC. Our findings revealed that the presence of a THC was associated with a significantly lower 6-month patency rate than AVF alone (78.20% vs 87.04%, *P* = .04). Furthermore, the multivariate analysis showed that the presence of a THC was an independent predictor of reduced patency (OR 0.49, 95% CI: 0.25–0.95, *P* = .03). These results suggest that the concurrent use of a THC with an AVF or AVG may negatively affect the patency of the vascular access, highlighting the importance of judicious use of THCs in patients undergoing HD.

The lower patency rate in the AVF + THC group suggests that the addition of a THC might have some adverse effects on AVF patency. Previous studies have reported a high incidence of central vein stenosis and decreased AVF survival in patients with prior dialysis catheter use.^[15-18]^ The exact mechanisms need further exploration; however, potential reasons could include increased vascular manipulation during surgery or altered blood flow dynamics.^[19,20]^ A plausible hypothesis is that catheter-induced stenosis of the central vein that serves as an outflow tract for the AVF may lead to hemodynamic changes that preclude AVF maturation or induce its early thrombosis.^[21,22]^

We also assessed functional dialysis success in the 2 groups and found no significant difference at 6 months (87.96% vs 83.89%, *P* = .21). This finding suggests that the presence of a THC may not significantly affect the short-term functional outcomes of vascular access, despite its association with reduced patency rates.

The divergence between these 2 endpoints warrants explanation. Primary patency and functional dialysis success are not interchangeable measures. Primary patency was considered lost whenever any intervention was required to maintain or restore flow, whereas functional dialysis success reflected only whether dialysis could ultimately be delivered through the access. Accesses in the THC group that developed stenosis were frequently salvaged by timely angioplasty or surgical revision; such an access loses primary patency yet remains usable for dialysis. Moreover, the presence of a functioning THC permitted uninterrupted dialysis while the access was being investigated and treated, removing the clinical pressure that might otherwise lead to premature access abandonment. Finally, the study was powered for the patency endpoint, and the 4.07% absolute difference in functional dialysis success (87.96% vs 83.89%) may reflect a type II error rather than true equivalence. Taken together, these observations suggest that concurrent THC use increases the need for interventions to maintain the access, rather than compromising the short-term delivery of dialysis itself.

This study identified several factors that were associated with a lower likelihood of functional dialysis success, including older age, use of a cubital artery to an autologous vein or graft, and higher Hb levels. Older age has been previously reported as a risk factor for complications and poor outcomes in patients undergoing HD.^[23]^ This may be attributed to the increased prevalence of comorbidities, reduced vascular quality, and impaired healing capacity in older individuals.^[24,25]^ The use of a cubital artery to an autologous vein or graft was also associated with a lower likelihood of functional dialysis success than a radial artery to an autologous vein. This finding is consistent with previous studies that have reported higher complication rates and lower patency rates for upper arm fistulas and grafts than for forearm fistulas.^[26,27]^

The incidence of access-related infection within 6 months was low and virtually identical between groups (5.56% without THC vs 5.21% with THC, *P* = .33), indicating that concurrent placement of a tunneled catheter was not associated with an excess of early access infection in this cohort. Given the small number of events, we did not pursue multivariable modeling of this endpoint, as any such analysis would have been substantially underpowered and the resulting estimates unstable. Infection following vascular access creation is nonetheless an important outcome, and the well-documented excess risk associated with prosthetic grafts relative to native fistulas warrants continued attention in clinical practice.^[28]^ Adequately powered studies with longer follow-up will be required to characterize the relationship between concurrent catheter use and access infection.

Furthermore, our multivariate analysis suggests that higher Hb levels may negatively affect 6-month AVF patency and functional dialysis success. Higher Hb levels might be associated with increased blood viscosity,^[29,30]^ potentially affecting AVF health. This finding underscores the importance of closely monitoring Hb levels in patients undergoing HD. In vascular access, the choice between grafts and radial–cephalic fistulas has been a subject of extensive investigation.^[31]^ The ORs clearly indicate that the type of vascular access plays a pivotal role in determining patency and availability. This is not a new revelation. A consensus has emerged in the medical community, backed by numerous studies,^[32-35]^ all pointing in the same direction: fistulas consistently outperform grafts in terms of outcomes. This collective understanding underscores the importance of informed decision-making in vascular access procedures.

This study has several limitations to consider when interpreting the results. First, the retrospective design may introduce potential confounders and limit the ability to establish causal relationships between the variables. Despite our efforts to account for multiple variables in the multivariate analysis, the potential for unmeasured confounders cannot be eliminated. Second, and most importantly, allocation of a THC was driven by clinical indication rather than by chance. Patients receiving a THC were more frequently referred late and are likely to have had poorer peripheral vasculature, a difference partially reflected in their significantly lower preoperative eGFR (7.88 vs 9.59 mL/min/1.73 m^2^, *P* = .03). Multivariable adjustment cannot fully remove this indication bias. The association between THC use and reduced patency should therefore be interpreted as identifying a higher-risk clinical phenotype rather than as evidence of a direct causal effect of the catheter itself.

Third, our database did not record the duration of catheter dwell, the date of catheter removal, or the interval from access creation to first cannulation. These variables would have allowed us to examine whether the duration of catheter exposure modifies the association with access patency, and their absence is a meaningful limitation of the present analysis. Prospective collection of these timepoints should be a priority in future work.

Fourth, the 6-month follow-up window was chosen because access maturation, catheter removal, and early access failure all occur within this interval. It is nonetheless insufficient to characterize long-term secondary patency, the cumulative burden of maintenance interventions, or catheter-related central venous stenosis, which typically becomes clinically manifest over a longer horizon. Our results should therefore be regarded as describing early access outcomes only.

Finally, the single-center design and the relatively modest sample size limit both external validity and statistical power. A subgroup analysis stratifying the THC group by access type would be informative since arteriovenous fistulas and grafts differ in hemodynamic behavior and complication profile; however, only 24 grafts were created within the THC group, which is insufficient for a stratified multivariable model. We note that graft use was an independent predictor of reduced patency in the overall cohort, suggesting that the combination of a graft and a concurrent THC may represent a particularly vulnerable configuration – a hypothesis requiring confirmation in an adequately powered multicenter cohort. Large-sample prospective, multicenter studies are needed to validate our findings.

The results of this study have significant ramifications for clinical practice and choices for the care of patients undergoing HD. The association between THC use and reduced patency rates highlights the need for careful consideration and judicious use of THCs in patients requiring vascular access. Strategies for optimizing vascular access and minimizing THC use may include early referral of patients with advanced CKD to a vascular access team, regular monitoring of vascular access function, and prompt intervention for access-related complications.^[36]^ Patient education and engagement in decision-making are also crucial to ensure informed consent and adherence to the recommended vascular access plan.^[37]^

## 5. Conclusion

This retrospective cohort study provides insights into the effect of tunneled hemodialysis catheters on the early outcomes of arteriovenous accesses. Concurrent THC use was independently associated with reduced 6-month primary patency, highlighting the importance of judicious catheter use and the timely creation of permanent vascular access. Notably, this reduction in patency was not accompanied by a significant reduction in functional dialysis success, suggesting that the principal consequence of concurrent catheter use is an increased need for interventions to maintain the access rather than a failure to deliver dialysis. We also identified graft use, older age, and higher hemoglobin levels as factors associated with poorer outcomes. This study has important limitations, including its retrospective single-center design, the 6-month follow-up window, and the indication bias inherent in catheter allocation; the associations reported here should therefore be regarded as hypothesis-generating. Prospective multicenter studies with longer follow-up are needed to validate these findings and to clarify the long-term effect of concurrent catheter use on vascular access outcomes.

## Acknowledgments

The authors thank Chih-I Chen, MD, of the Division of Colon and Rectal Surgery, E-Da Hospital, who served as principal investigator of record for the institutional review board protocol under which this study was conducted. Dr Chen did not participate in study design, data analysis, or manuscript preparation and does not meet the ICMJE criteria for authorship.

## Author contributions

**Conceptualization:** Heng Su, Hsuan-Yin Wu.

**Data curation:** Heng Su, Hsuan-Yin Wu.

**Formal analysis:** Hsuan-Yin Wu.

**Investigation:** Heng Su, Ting-Wei Lin.

**Methodology:** Ting-Wei Lin, Jian-Han Chen.

**Project administration:** Ting-Wei Lin, Jian-Han Chen.

**Supervision:** Hsuan-Yin Wu.

**Writing – original draft:** Heng Su.

**Writing – review & editing:** Heng Su, Ting-Wei Lin, Jian-Han Chen, Hsuan-Yin Wu.

## References

[1] Schena FP. Epidemiology of end-stage renal disease: international comparisons of renal replacement therapy. Kidney Int. 2000;57:S39–45.

[2] Lai T-S Hsu C-C Lin M-H Wu V-C Chen Y-M. Trends in the incidence and prevalence of end-stage kidney disease requiring dialysis in Taiwan: 2010-2018. J Formos Med Assoc. 2022;121(suppl 1):S5–S11. 10.1016/j.jfma.2021.12.013 35074236

[3] National Health Insurance Administration. National Health Insurance Annual Report. MoHaW, ROC; 2020.

[4] Schmidli J Widmer MK Basile C. Editor’s choice–vascular access: 2018 clinical practice guidelines of the European Society for Vascular Surgery (ESVS). Eur J Vasc Endovasc Surg. 2018;55:757–818. 10.1016/j.ejvs.2018.02.001 29730128

[5] Shechter SM Skandari MR Zalunardo N. Timing of arteriovenous fistula creation in patients with CKD: a decision analysis. Am J Kidney Dis. 2014;63:95–103. 10.1053/j.ajkd.2013.06.021 23978336

[6] Hayashi R Huang E Nissenson AR. Vascular access for hemodialysis. Nat Clin Pract Nephrol. 2006;2:504–13. 10.1038/ncpneph0239 16941043

[7] Jenkins GW Kelly M Anwar S Ahmed SS. The S-Tunnel for tunnelled dialysis catheter: an alternative approach to the prevention of displacement. J Vasc Access. 2015;16:527–9. 10.5301/jva.5000455 26349889

[8] Lok CE Huber TS Lee T; National Kidney Foundation. KDOQI clinical practice guideline for vascular access: 2019 update. Am J Kidney Dis. 2020;75(4 suppl 2):S1–S164. 10.1053/j.ajkd.2019.12.001 32778223

[9] Almasri J Alsawas M Mainou M. Outcomes of vascular access for hemodialysis: a systematic review and meta-analysis. J Vasc Surg. 2016;64:236–43. 10.1016/j.jvs.2016.01.053 27345510

[10] Vascular Access 2006 Work Group. Clinical practice guidelines for vascular access. Am J Kidney Dis. 2006;48(suppl 1):S176–247. 10.1053/j.ajkd.2006.04.029 16813989

[11] Celik S Oguz EG Okyay GU Selen T Ayli MD. The impact of arteriovenous fistulas and tunneled cuffed venous catheters on morbidity and mortality in hemodialysis patients: a single center experience. Int J Artif Organs. 2021;44:229–36. 10.1177/0391398820952808 32962489

[12] Tamura MK Tan JC O’hare AM. Optimizing renal replacement therapy in older adults: a framework for making individualized decisions. Kidney Int. 2012;82:261–9. 10.1038/ki.2011.384 PMC3396777 22089945

[13] Sidawy AN Spergel LM Besarab A; Society for Vascular Surgery. The Society for Vascular Surgery: clinical practice guidelines for the surgical placement and maintenance of arteriovenous hemodialysis access. J Vasc Surg. 2008;48:2S–25S. 10.1016/j.jvs.2008.08.042 19000589

[14] Lee T Barker J Allon M. Comparison of survival of upper arm arteriovenous fistulas and grafts after failed forearm fistula. J Am Soc Nephrol. 2007;18:1936–41. 10.1681/ASN.2006101119 17475812

[15] Wu C-K Huang Y-C Lin C-H Chen M. Bridge of tunneled cuffed catheter as a risk for future arteriovenous fistulae failure. J Clin Med. 2022;11:1289. 10.3390/jcm11051289 PMC8911096 35268379

[16] Rizvi SA Usoh F Hingorani A. The clinical efficacy of balloon-assisted maturation of autogenous arteriovenous fistulae. Ann Vasc Surg. 2017;41:41–5. 10.1016/j.avsg.2016.08.022 27903478

[17] Peterson WJ Barker J Allon M. Disparities in fistula maturation persist despite preoperative vascular mapping. Clin J Am Soc Nephrol. 2008;3:437–41. 10.2215/CJN.03480807 PMC2390953 18235150

[18] Rayner HC Pisoni RL Gillespie BW; Dialysis Outcomes and Practice Patterns Study. Creation, cannulation and survival of arteriovenous fistulae: data from the dialysis outcomes and practice patterns study. Kidney Int. 2003;63:323–30. 10.1046/j.1523-1755.2003.00724.x 12472799

[19] Poinen K Quinn RR Clarke A. Complications from tunneled hemodialysis catheters: a Canadian observational cohort study. Am J Kidney Dis. 2019;73:467–75. 10.1053/j.ajkd.2018.10.014 30642607

[20] Agarwal AK Patel BM Haddad NJ. Central vein stenosis: A nephrologist’s perspective. In: Seminars in Dialysis. Wiley Online Library, 2007. 10.1111/j.1525-139X.2007.00242.x 17244123

[21] Shingarev R Barker-Finkel J Allon M. Association of hemodialysis central venous catheter use with ipsilateral arteriovenous vascular access survival. Am J Kidney Dis. 2012;60:983–9. 10.1053/j.ajkd.2012.06.014 PMC4017344 22824354

[22] Salgado OJ Urdaneta B Colmenares B García R Flores C. Right versus left internal jugular vein catheterization for hemodialysis: complications and impact on ipsilateral access creation. Artif Organs. 2004;28:728–33. 10.1111/j.1525-1594.2004.07316.x 15270954

[23] Lee H-J Son Y-J. Prevalence and associated factors of frailty and mortality in patients with end-stage renal disease undergoing hemodialysis: a systematic review and meta-analysis. Int J Environ Res Public Health. 2021;18:3471. 10.3390/ijerph18073471 PMC8037521 33801577

[24] Song Y-H Cai G-Y Xiao Y-F Chen X-M. Risk factors for mortality in elderly haemodialysis patients: a systematic review and meta-analysis. BMC Nephrol. 2020;21:377. 10.1186/s12882-020-02026-x PMC7457491 32867718

[25] Chen J Wang J Liu Y. Mortality and associated risk factors between young and elderly maintenance haemodialysis patients: a multicentre retrospective cohort study in China. BMJ Open. 2023;13:e066675. 10.1136/bmjopen-2022-066675 PMC9906252 36746548

[26] Fitzgerald JT Schanzer A McVicar JP Chin AI Perez RV Troppmann C. Upper arm arteriovenous fistula versus forearm looped arteriovenous graft for hemodialysis access: a comparative analysis. Ann Vasc Surg. 2005;19:843–50. 10.1007/s10016-005-7419-y 16177869

[27] Chue KM Thant KZ Luo HD Soh YHR Ho P. Comprehensive comparison of the performance of autogenous brachial-basilic transposition arteriovenous fistula and prosthetic forearm loop arteriovenous graft in a multiethnic Asian hemodialysis population. Biomed Res Int. 2016;2016:8693278. 10.1155/2016/8693278 PMC5093232 27840832

[28] Stevenson KB Hannah EL Lowder CA. Epidemiology of hemodialysis vascular access infections from longitudinal infection surveillance data: predicting the impact of NKF-DOQI clinical practice guidelines for vascular access. Am J Kidney Dis. 2002;39:549–55. 10.1053/ajkd.2002.31405 11877574

[29] Ho C-H. White blood cell and platelet counts could affect whole blood viscosity. J Chin Med Assoc. 2004;67:394–7. 15553798

[30] Strazhesko I Staroverova A Borisov E Orlova Y. Red blood cells count and hemoglobin level are associated with arterial stiffness in healthy subjects. J Hypertens. 2018;36(suppl 1):e250–1.

[31] Lazarides MK Georgiadis GS Antoniou GA Staramos DN. A meta-analysis of dialysis access outcome in elderly patients. J Vasc Surg. 2007;45:420–6. 10.1016/j.jvs.2006.10.035 17264030

[32] Manns B Tonelli M Yilmaz S. Establishment and maintenance of vascular access in incident hemodialysis patients: a prospective cost analysis. J Am Soc Nephrol. 2005;16:201–9. 10.1681/ASN.2004050355 15563567

[33] Lyu B Chan MR Yevzlin AS Astor BC. Catheter dependence after arteriovenous fistula or graft placement among elderly patients on hemodialysis. Am J Kidney Dis. 2021;78:399–408.e1. 10.1053/j.ajkd.2020.12.019 33582176

[34] Lok CE Sontrop JM Tomlinson G. Cumulative patency of contemporary fistulas versus grafts (2000-2010). Clin J Am Soc Nephrol. 2013;8:810–8. 10.2215/CJN.00730112 PMC3641610 23371955

[35] Lee T Thamer M Zhang Q Zhang Y Allon M. Vascular access type and clinical outcomes among elderly patients on hemodialysis. Clin J Am Soc Nephrol. 2017;12:1823–30. 10.2215/CJN.01410217 PMC5672965 28798220

[36] Ibeas J Roca-Tey R Vallespín J; por el Grupo Español Multidisciplinar del Acceso Vascular (GEMAV). Spanish clinical guidelines on vascular access for haemodialysis. Nefrologia. 2017;37(suppl 1):1–191. 10.1016/j.nefro.2017.11.004 29248052

[37] Liu J Zhou Y Tang Y Chen J Li J. Patient engagement during the transition from nondialysis‐dependent chronic kidney disease to dialysis: a meta‐ethnography. Health Expect. 2023;26:2191–204. 10.1111/hex.13850 PMC10632643 37641530

